# P-391. Impact of Rapid Antiretroviral Therapy Initiation on Retention in Care and Viral Suppression in an Urban HIV Clinic in Louisville, Kentucky

**DOI:** 10.1093/ofid/ofaf695.608

**Published:** 2026-01-11

**Authors:** T’shura Ali, Forest W Arnold, Vidyulata Salunkhe, Steven Gootee, Lucia Puga Sanchez, Deepti Deepti, Aleena Naeem, Rana Anwar, Bailey Benidir

**Affiliations:** University of Louisville School of Medicine, Louisville, KY; University of Louisville School of Medicine, Louisville, KY; University of Louisville School of Medicine, Louisville, KY; University of Louisville School of Medicine, Louisville, KY; University of Louisville School of Medicine, Louisville, KY; University of Louisville, Louisville, Kentucky; University of Louisville, Louisville, Kentucky; University of Louisville, Louisville, Kentucky; University of Louisville School of Medicine, Louisville, KY

## Abstract

**Background:**

Successful treatment of HIV depends on testing, time to antiretroviral therapy (ART), and retention in care. The objective of this study was to determine if rapid initiation of ART was associated with increased retention in care and viral suppression in newly diagnosed HIV patients.

Time to Undetectable Viral Load in the First Year of Care Compared across Rapid and Non-Rapid ART

**Figure:**
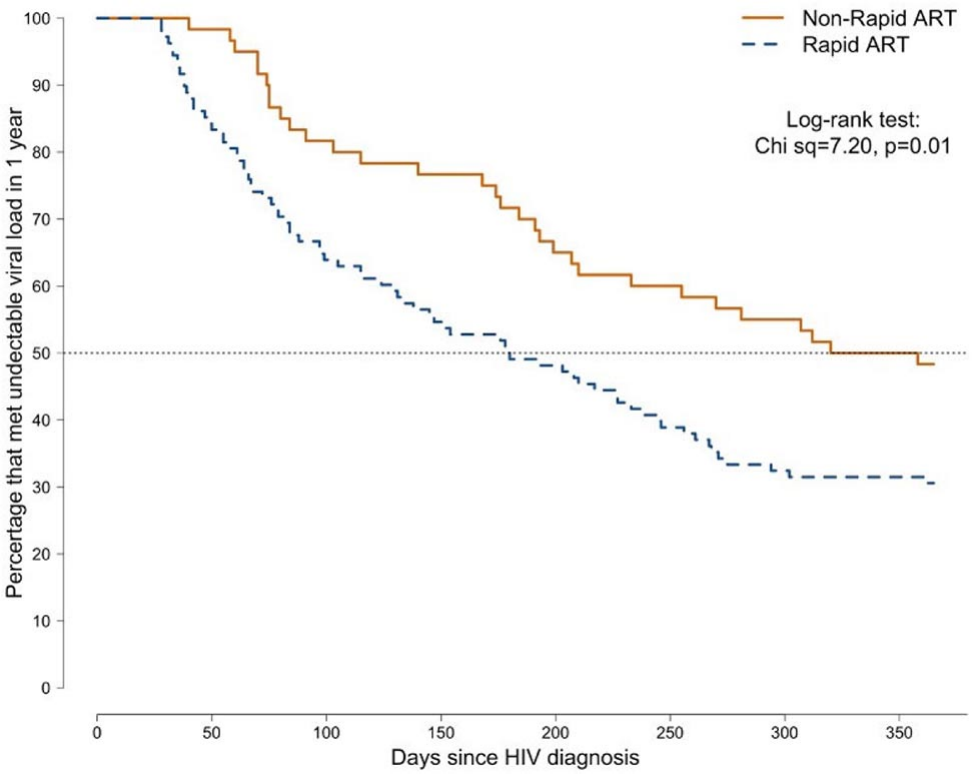

**Methods:**

This was a prospective cohort study of patients newly diagnosed with HIV seeking care at an HIV clinic between July 1, 2021, through June 30, 2024. All patients were ≥ 18 years old and treatment-naїve. Patients who received treatment within seven days of diagnosis were enrolled into the rapid arm; all other participants were enrolled into the non-rapid arm. Patients were then followed for a period of 15 months of care. Short- and long-term outcomes assessed included retention and time to undetectable viral load. The time to undetectable viral load was compared using Log-rank tests using Kaplan-Meier curves. Multivariable logistic regression and cox proportional hazards regression was used to examine the association between rapid ART and HIV care outcomes. Odds ratios (OR) and hazards ratios (HR) were reported respectively, along with the 95% confidence intervals.

**Results:**

Of 168 treatment-naїve patients, 108 (64%) were eligible for the rapid arm and 60 (36%) for the non-rapid arm. Nearly all patients were treated with bictegravir/emtricitabine/tenofovir alafenamide (BIC/FTC/TAF). Non-rapid arm patients were diagnosed on average 38 days prior to presenting to clinic. Retention rates were significantly higher for the rapid arm after 3-4 months; (50% *vs* 27%; *P*=0.006), but not after one year; (43% *vs* 40%; *P*=0.87). A higher proportion of rapid ART patients reached an undetectable viral load after one year from HIV diagnosis; (69% *vs* 52%; *P*=0.03). The time to reach an undetectable viral load was also significantly shorter in the rapid ART arm; (180 days *vs* 339 days, *P*=0.02).

**Conclusion:**

The current study found that patients newly diagnosed with HIV who received rapid ART had the benefit of short-term retention (3 months) and long-term viral load suppression (1 year) with a shorter time to reach undetectable. The results of this study contribute to a knowledge gap and may assist in determining how to direct available funds to assist patients.

**Disclosures:**

T'shura Ali, PhD, MPH, Gilead Sciences: Grant/Research Support Forest W. Arnold, DO, MSc, Gilead Sciences: Grant/Research Support Vidyulata Salunkhe, MBBS, MPH, Gilead Sciences: Grant/Research Support Steven Gootee, MHI, Gilead Sciences: Grant/Research Support Lucia Puga Sanchez, MD, Gilead Sciences: Grant/Research Support Deepti Deepti, MPH, Gilead Sciences: Grant/Research Support Aleena Naeem, MD, Gilead Sciences: Grant/Research Support Rana Anwar, MD, Gilead Sciences: Grant/Research Support Bailey Benidir, PharmD, AAHIVP, Gilead Sciences: Grant/Research Support

